# Growth and age differences between two male alternative reproductive tactics in the plainfin midshipman

**DOI:** 10.1111/jfb.70274

**Published:** 2025-11-17

**Authors:** Micah J. Quindazzi, Angus Townsend, Aneesh P. H. Bose, Sigal Balshine, Francis Juanes

**Affiliations:** ^1^ Department of Biology University of Victoria Victoria British Columbia Canada; ^2^ Department of Wildlife, Fish, and Environmental Studies Swedish University of Agricultural Sciences Umeå Sweden; ^3^ Department of Psychology, Neuroscience & Behaviour McMaster University Hamilton Ontario Canada

**Keywords:** ageing, Batrachoididae, life history, ontogeny, otoliths, *Porichthys notatus*, toadfish, von‐Bertalanffy growth function

## Abstract

The plainfin midshipman (*Porichthys notatus* Girard, 1854) is a toadfish with two distinct reproductive male tactics: ‘guarder males’ and ‘sneaker males’. These two tactics are anatomically, physiologically and behaviourally distinct from one another at sexual maturity, but it remained unclear whether these two male types remain fixed or plastically transition from one to another across a lifetime. To examine this question, we aged the sagittal otoliths (inner ear bones) of 371 adult plainfin midshipman via the break‐and‐burn method. Our study showed that guarder males were on average larger and older (mean age: 7.0 years, range: 3–17) than sneaker males (mean age: 3.4 years, range: 2–8) and females (mean age: 5.1 years, range: 2–10). There was considerable overlap in age between guarder and sneaker males; the two tactics were of similar body sizes at age 3, but after that guarder males were always bigger. We used a two‐factor von Bertalanffy growth functions (VBGFs) to show that males versus females and guarder versus sneaker males had divergent growth trajectories. The VBGF estimates and distance measures from the otolith centre to the first annual otolith mark suggest that sneakers initially grew more quickly. Our results support the hypothesis that the two male reproductive tactics in plainfin midshipman likely represent distinct fixed life‐history pathways that differ early in their development.

## 1 INTRODUCTION

Alternative reproductive tactics (ARTs) are a taxonomically widespread phenomenon in which same‐sex conspecifics attempt to achieve reproduction through alternative means. ARTs often occur in males when fitness can be gained by exploiting others' reproductive investments (Oliveira et al., 2008) and are typically characterized by discontinuous within‐sex variation in morphology, physiology and/or behaviour. In some species, the same individuals will employ different ARTs, switching rapidly back and forth, depending on the environmental or physiological conditions they find themselves in (known as ‘conditional’ or ‘plastic’ tactics) (Gross, 1996). In other species, ARTs are less flexible, with individuals either specializing and adopting one tactic over their entire lifetimes (‘fixed’ tactics) or transitioning from one tactic to another one only as they grow older, larger or more experienced (‘sequential’ tactics) (Taborsky et al., 2008). Age and growth studies can aid in differentiating between fixed and sequential tactics, for example, by revealing whether males adopting the different reproductive tactics comprise (i) distinct age classes that follow a single growth trajectory (indicative of a sequential tactic), or (ii) potentially overlapping age classes that follow separate growth trajectories (indicative of a fixed tactic).

Alternative reproductive tactics are common among fishes because most fish have indeterminate growth, which exaggerates the competitive advantage enjoyed by large males (Engqvist & Taborsky, 2016; Taborsky, 1999). Because many fish species also have male paternal care and external fertilization, there is fitness to be exploited by adopting a parasitic reproductive strategy that takes advantage of conventional male's reproductive investment in courtship, territoriality and care (Taborsky, 1999, 2001, 2008). Some species show a complex combination of both fixed and sequential tactics. For example, the African cichlid, *Lamprologus callipterus*, shows a genetically induced, paternally inherited, fixed tactic expression. ‘Nesting’ males control territories with collections of empty snail shells that females use to lay eggs and raise offspring in, whereas ‘dwarf’ males steal fertilizations away from nesting males by hiding within the shells (Wirtz‐Ocaňa et al., 2013, Wirtz‐Ocaňa et al., 2014). However, before nesting male *L. callipterus* are large and old enough to control territories, they adopt a ‘sneaker’ tactic that reproduces by opportunistically intruding on spawning events between nesting males and females (von Kuerthy & Taborsky, 2016). Another example is seen in male Mediterranean wrasse, *Symphodus ocellatus*, which can display three male ARTs, ‘nesting’, ‘satellite’ or ‘sneaker’ males, with individuals transitioning between tactics either from sneaker to satellite tactic, or from satellite to nesting tactic between their first and second years of life (Alonzo et al., 2000; Taborsky, 1987). Often ARTs have distinct age‐growth trajectories (Wirtz‐Ocaňa et al., 2013, 2014; Taborsky, 1998, Taborsky, 2008). Individuals born earlier will have more time to grow, which, in turn, can impact reproductive tactic adoption, a phenomenon known as a ‘birthdate effect’ (Taborsky, 1998; Welsh et al., 2017). Across species, one common pattern observed is that males adopting the sneaker/parasitic tactic tend to be smaller, and guarder/nesting/courting males tend to be larger (Alonzo et al., 2000; Gross, 1991; Magnhagen & Kvarnemo, 1989; Smith et al., 2023; Wirtz‐Ocaňa et al., 2013), though ageing and growth data are imperative to uncover the true ontogeny of many ARTs.

The plainfin midshipman, *Porichthys notatus* (Batrachoididae), is a species of marine toadfish with two strikingly different male reproductive morphs, called ‘guarder’ (or ‘Type I') males and ‘sneaker’ (or ‘Type II') males, respectively (Bass et al., 1996; Bose et al., 2016, 2020; Brantley & Bass, 1994; Cogliati, Balshine, & Neff, 2014, Cogliati, Mistakidis, et al., 2014). Guarder males are large, aggressive, nest builders that attract females through high‐frequency vibrations of sonic muscles on their swim bladder and provide extended paternal care for offspring (Bose et al., 2018; Brantley & Bass, 1994; Fitzpatrick et al., 2016). In contrast, sneaker males are small, do not vocalize and do not provide parental care, but instead invest heavily into traits related to sperm competition to cuckold guarder males (Brantley & Bass, 1994; Cogliati, Balshine, & Neff, 2014, Cogliati, Mistakidis, et al., 2014; Fitzpatrick et al., 2016; Miller et al., 2019). Sexually mature guarder males have larger and more connected vocal‐motor brain neurons, and much larger and more vascularized sonic muscles needed to vocalize compared to sneaker males (Bass et al., 1996; Brantley et al., 1993). Neuroanatomical differences related to vocal circuitry are already differentiated between the tactics at their sexual maturity (Bass et al., 1996), implying that the two male tactics are different developmental trajectories of a ‘fixed’ strategy (Taborsky, 2008). Further evidence suggests that this differentiation is determined by a developmental threshold, possibly one based on social cues or body condition at an early stage of development, because rearing density of juveniles can influence the proportion of males that adopt each tactic at maturity (Foran, 1998). However, because Bass et al. (1996) did not age fish across their lifetimes, the possibility remains that sneaker males eventually transition to become guarder males later in life and thus could also display a ‘sequential’ progression. To clarify this, using otoliths (inner ear bones) we aged guarder, sneaker and female plainfin midshipman fish captured from the wild. More specifically, we had two research aims: (1) to determine age and assess age differences between the sexes and between guarder and sneaker males; and (2) to use size at age, estimated growth functions and growth intervals indicated by otolith marks to evaluate growth differences between the sexes and between guarder and sneaker males.

## 2 METHODS

Adult plainfin midshipman (*n* = 374) were collected at low tide from various intertidal nesting sites around the Salish Sea between 2011 and 2023 (Table S1 provides the exact location of all the study collection sites and the numbers of fish collected per site). All the fish used in this study were previously used in many other studies (Bose et al., 2014, 2016, Bose et al., 2017, 2018, 2020; Brown et al., 2023; Cogliati, Balshine, & Neff, 2014; Cogliati et al., 2015; Cogliati, Mistakidis, et al., 2014; Halliday et al., 2018; Harrison‐Weiss et al., 2025; Townsend, 2023). To collect fish, we gently lifted rocks and when we discovered a nest with eggs or hatched young adhered on the nest roof, we collected the guarder male as well as any females and sneaker males also in the nest (see Appendix S1 and Bose et al., 2018; Cogliati et al., 2015 for additional details on field sampling and site information).

Animal collection and handling followed McMaster University's Animal Care protocols (AUP: Balshine 2013‐11‐07; 2018‐01‐02; 2022‐03‐06), as derived from standards established by the Canadian Council on Animal Care (Olfert et al., 1993). All fish collected were euthanized in a bath of benzocaine or MS‐222 followed by cervical severance. Each fish was weighed for body mass (to the nearest 0.1 g) and measured for standard length (SL, to the nearest millimetre) before dissection and removal of their saccular otoliths. The sex of each adult fish was confirmed by internal inspection of their gonads, and male tactic was determined based on a combination of traits, including the relative testes size, the relative size of the accessory gland, the shape of the swim bladder and the size and colour of the sonic muscle attached to the swim bladder (Bass et al., 1996; Mohr et al., 2017). Guarder males have relatively small testes that are attached to large accessory glands and hypertrophied, red sonic muscles that surround a very rounded swim bladder. In contrast, sneaker males have relatively large testes with small accessory glands, and small, pale pink or white sonic muscles on conical‐shaped swim bladders (Bass et al., 1996; Bass & Marchaterre, 1989; Mohr et al., 2017; Rogers et al., 2023). We extracted, cleaned and stored dry each fish's saccular otoliths (inner ear bones, found on either side of the fish brain). In total, we aged the otoliths from 176 guarder males, 138 females and 57 sneaker males in this study (though 3 fish had to eventually be excluded due to damaged otoliths). We intentionally used the otoliths from fish representing the full range of adult fish body sizes for the different tactics and sexes, including some of the smallest and largest guarder males, sneaker males and females collected in the Salish Sea.

**FIGURE 1:**
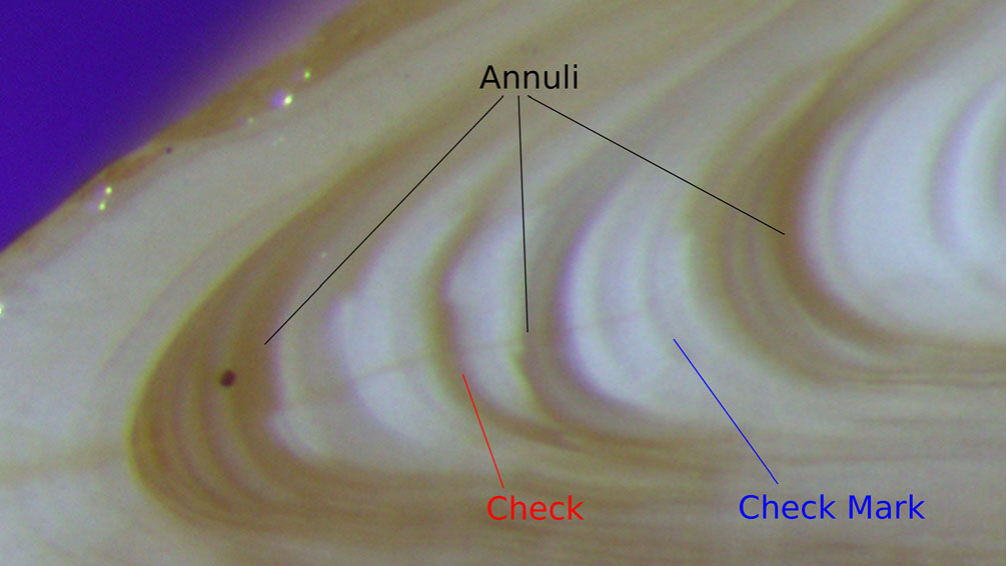
Zoomed in image of a transverse cross‐section through the sagittal (i.e., saccular) otoliths of plainfin midshipman (*Porichthys notatus*). Annuli are highlighted in black and are identified by the strength of the mark left after the break‐and‐burn method and the regular intervals they occur at. Checks (red) are strong marks like annuli but appear at irregular intervals. Check marks (blue) are weaker than checks or annuli and appear at irregular intervals.

In the laboratory, the otoliths were further cleaned to remove any leftover soft tissue through sonication in a deionized water‐bath. In a pilot study, we validated which ageing technique works best for plainfin midshipman (Townsend, 2023) and then implemented the most successful one, a modified break‐and‐burn method (Christensen, 1964), on all the saccular otoliths from guarder male, sneaker male and female plainfin midshipman fish that we had collected from the Salish Sea (Canada, USA). In brief, otoliths were transversely bisected through the core using a Buehler IsoMet Low Speed Precision Cutter (Buehler, Lake Bluff, Illinois; https://www.buehler.com/). One half was burned in an alcohol flame until a sufficient yellow‐brown colouring was achieved (Figure 1). The otoliths were then photographed using an Olympus SZX16 stereoscope, DP26 camera and CellSens Standard software (Olympus, Shinjuku, Tokyo; www.olympus-global.com). Annuli (the periodic zones of translucent and opaque rings associated with seasonal changes in growth rate over the course of a year) were identified by the appearance of strong, continuous, dark bands at regular intervals through the transverse plane.

In addition to the annuli on the otoliths, we could clearly distinguish checks (zones of translucent and opaque rings that appear distinct but at irregular intervals) and check marks (zones of translucent and opaque rings that are weaker than checks and observed at irregular intervals) by their strength and regularity of the mark (Figure 1). Sometimes annuli preceded and/or succeeded a check, and these were marked collectively as one singular annulus. To identify the relative growth per year for guarders, sneakers and females, distances were measured from the centre of the otolith (the primordia) along the ventral midline to the start of the detachment check and to the first three annuli. These distances were taken from otoliths that were soaked from 24 h in deionized water to clear up the annuli in the image as the transverse cuts in the otoliths were too variable in position to produce reliable distances that could be compared across individuals. These distances were measured in individuals ranging from 2 to 5 years old to ensure that all features would be easily discernible, as older fish have thicker otoliths, which obscure features in the middle of the otolith. This did not impact our ageing.

In addition to ageing adult otoliths, we examined the otoliths from three groups of juvenile plainfin midshipman, which helped validate our growth modelling and ageing. First, we collected 15 young‐of‐the‐year (YOY) near the end of the spawning season that had just newly detached from their rock surface (~2 months after oviposition, Cogliati et al., 2013). These juveniles collected from nests at Transfer Beach, Ladysmith, British Columbia, on 27 July 2022 were on average 2.04 ± 0.03 cm long (range: 1.78–2.43 cm SL). Second, we also collected 15 age 1+ fish by seining in the eelgrass beds on Thetis Island, British Columbia (48°59′N, 123°39′W), on 19 June 2022. These fish were presumed to be age 1+, as they were on average 6.46 ± 0.16 cm long (range: 5.60–7.88 cm SL), more than thrice the length of known recently detached YOY and were similar in size to midshipman reared for nearly 1 year in laboratory settings (Brown et al., 2023). Third, we examined 314 YOY fish that were born in the laboratory with known birthdates and detachment dates and reared between the ages of 0 to 279 days old post‐detachment (Brown et al., 2023, Figure 2). These fish were reared in similar conditions to wild midshipman (see Brown et al., 2023 for rearing details) and had reached a maximum size of 4.75 cm in captivity after 279 days post‐detachment.

**FIGURE 2:**
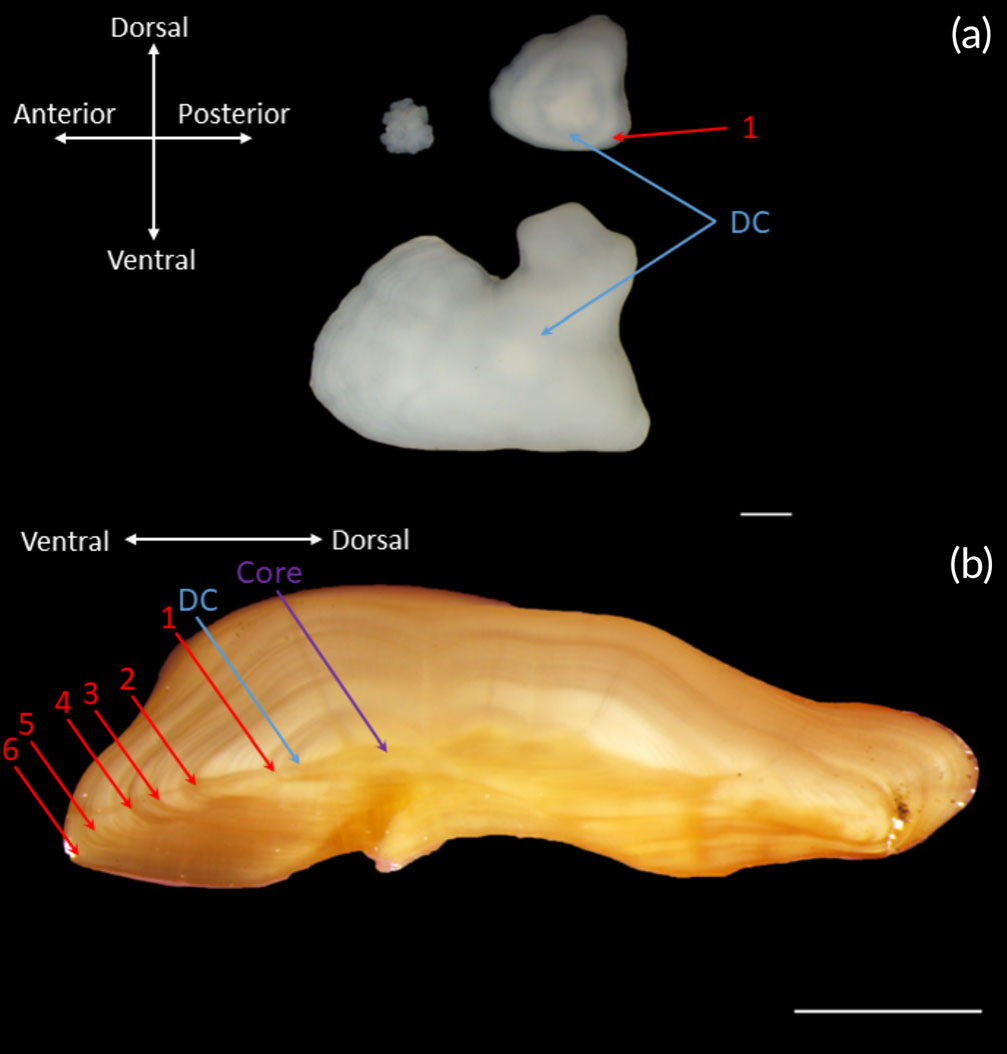
Sagittal (i.e., saccular) otoliths of plainfin midshipman (*Porichthys notatus*). (a) Sagittal otoliths of a young‐of‐the‐year (top left), 1‐year‐old juvenile (top right) and a 6‐year‐old adult guarder male (bottom). The detachment check (DC) is labelled on the juvenile and adult fish and indicated by the blue arrows. The first annulus is marked on the juvenile fish only (‘1’). Otoliths were soaked in deionized water for 4 h to improve visibility of microstructure. (b) Sagittal otolith of a guarder male plainfin midshipman, broken and burned, and then soaked in immersion oil. The core is marked by a purple arrow, and the DC is marked again with a blue arrow. Annuli are labelled with red arrows. Ageing was done through the ventral axis and directly under the microscope rather than from photographs. The estimated age of this individual was 6 years.

We identified the universal presence of a strong ‘detachment’ check ring that formed when the fish first detach from the substrate of the nest ceiling. This detachment check was clearly visually identified on all the otoliths of newly detached plainfin midshipman young (Campana, 1984, see Figure 2a). This visually distinctive otolith structure is likely related to growing upside down prior to detaching from the rock surface (Figure 2a).

von Bertalanffy growth functions (VBGFs) were used to model growth across time (von Bertalanffy, 1938; Equation 1). In this equation, *L*_*t*_ is the length at age *t*, *L*_∞_ is a function of the asymptotic maximum length, *L*_0_ is the length at age zero, k is the growth rate parameter and *t* is time (or age in years). Fisheries models typically assume the age of zero to be the date of settlement (i.e., when pelagic larvae settle onto a benthic habitat, e.g., Payet et al., 2020). Although in most three parameter VBGFs, *L*_0_ is estimated, we opted to use a two‐parameter VBGF to avoid issues with overestimating size at birth (Pardo et al., 2013, see Supporting Information for a comparison of the different growth functions). Because plainfin midshipman do not have pelagic larvae, we took the age of zero to be the day the juveniles detached from the nest surface and become free swimming. *L*_0_ was therefore fixed for all midshipman at the average length of midshipman YOY that had just detached from the nest ceiling (*L*_0_ = 2.035 cm). *L*_∞_ and k were estimated from the age and growth data using the nls function in R with the starting estimations of 20 cm and −0.1, respectively, split across the three types of midshipman encountered (guarders, sneakers and females). The theoretical age at a length of 0 (t_0_) was also calculated to calculate longevity, though it was not needed for the VBGF equations used in this study. Longevity (A_0.95_) was considered to be the estimated age at which a fish is expected to reach 95% of its maximum length *L*_∞_ (as determined by eq. 6 in Taylor (1958).

(1) $$L_{t}=L_{\infty }-\left(L_{\infty }-L_{0}\right)e^{-\mathrm{kt}}$$

We therefore obtained three VBGFs by fitting Equation (1) to our data on fish length and age for guarder males, sneaker males and females separately. These observed VBGFs were used to predict the size at age for each category of adult midshipman fish in our dataset, and they also provided us with a growth rate parameter, k, and *L*∞, unique to each sex and tactic.

### 2.1 Statistical analyses

All analyses were performed using R version 4.3.0 (R Core Team, 2023) and RStudio version 2023.06 (RStudio Team, 2023). Code and data are available as part of our Supporting Information. To find k for the VBGFs, we used the non‐linear least squares function from the base stats package in R (nls). The one‐dimensional root finding function (uniroot) from the base stats package in R was used to determine t_0_. A likelihood ratio test of nested models was used to determine if the VBGFs differed among the guarder males, sneaker males and females. To test whether these growth parameters differed significantly among sneakers, guarders and females, both k and *L*_∞_ were bootstrapped using the ordinary non‐parametric simulation with replacement in the boot command in R. These simulations were carried out 5000 times for each type of midshipman and the results were used to test whether the 95% confidence intervals overlapped.

Likelihood ratio tests of nested models were used to determine if length‐to‐weight relationships differed among guarder males, sneaker males or females. Permutation *t*‐tests from the MKinfer package in R (perm.t.test) were used to determine if fish differed in size at age within specific defined age classes, and a permutational multivariate analysis of variance (PERMANOVA) from the vegan package in R (adonis2) was used to identify if there were differences in average age across females, sneakers and guarders (Kohl, 2023; Oksanen et al., 2022).

## 3 RESULTS

**FIGURE 3:**
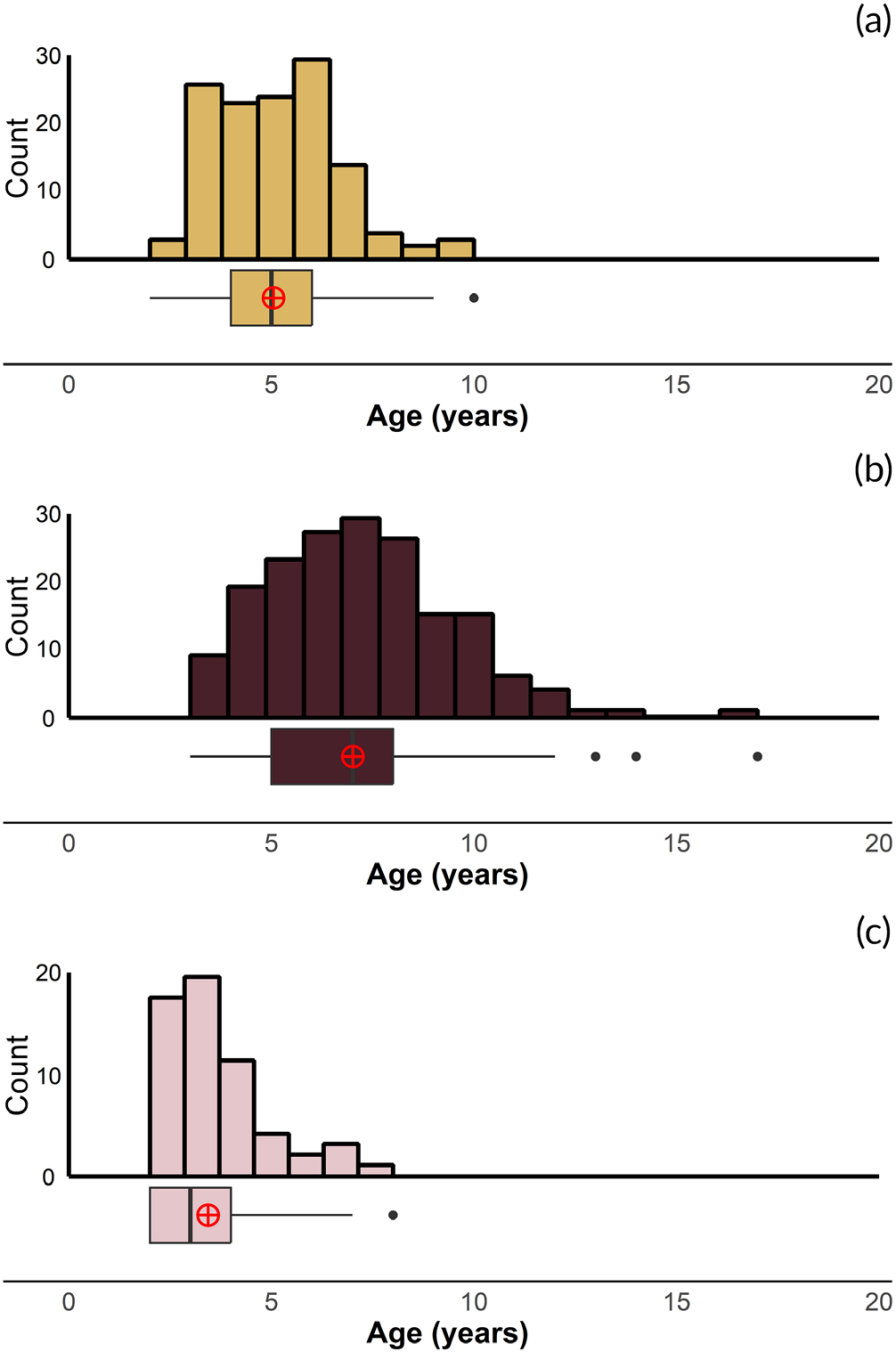
A combination histogram and boxplot showing the overall age distributions for *Porichthys notatus* females in yellow (a, *n* = 138), guarder males in dark maroon (b, *n* = 176) and sneaker males in light pink (c, *n* = 57). All fish in this comparison were collected from the wild (see Methods above). Black bars in the boxplot represent the *median age* of the group of same sex or same tactic individuals, whereas the red circles with the cross inside indicate the *mean age* for that sex or tactic.

The adult plainfin midshipman used in this study varied in age between 2 and 17 years, with the majority of fish being between the ages of 3 and 7 (Figure 3). Guarder males were the oldest on average (x¯ = 7.0, range: 3–17 years, PERMANOVA; F_2,368_ = 82.7, *p* < 0.001) and were the only class of fish found to be above 10 years of age (Figure 3). Females were the next oldest with an average age of x¯ = 5.1 years, range: 2–10 years, and sneaker males were the youngest class of plainfin midshipman with an average age of x¯ = 3.4 years, range: 2–8 years. Therefore, the only 2‐year‐olds sampled from the breeding ground were either sneaker males or females, with the smallest/youngest guarder males in our collection being 3‐year‐olds.

**FIGURE 4:**
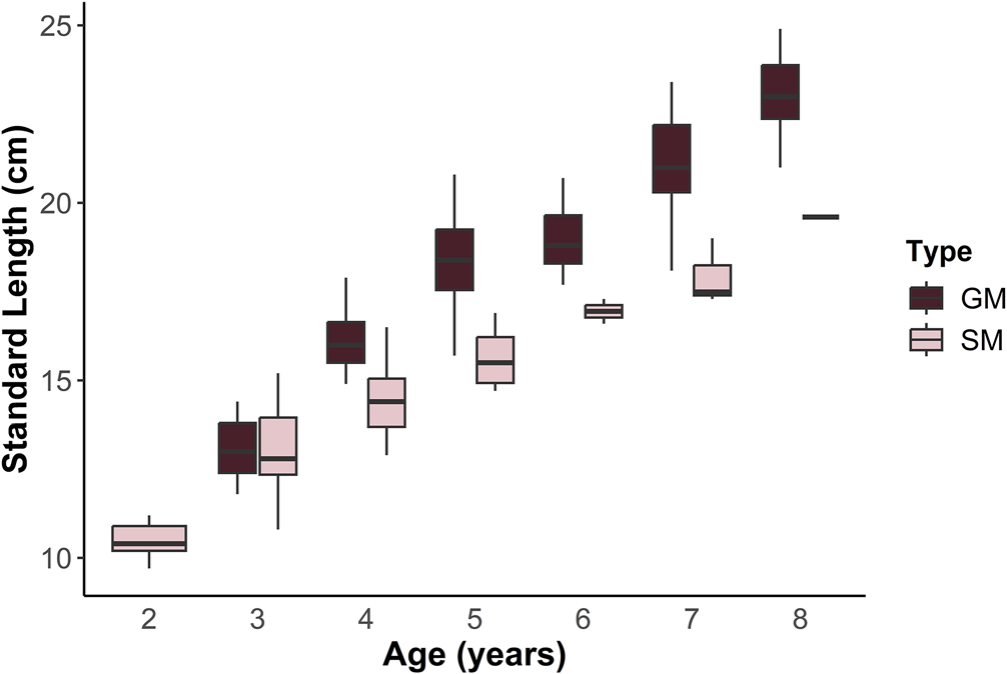
Boxplots showing the standard lengths of *Porichthys notatus* guarder males (GM, *n* = 176) and sneaker males (SM, *n* = 57) between ages 2 and 8. The black horizontal line represents the median value.

Body mass ranged from 10.3 to 371.4 g and SL from 9.7 to 29.5 cm, with guarder males being the heaviest (x¯ = 128.8 g, range: 19.2–371.4 g) and the longest fish (x¯ = 20.7 cm, range: 11.8–29.5 cm). Sneaker males were the lightest (x¯ = 34.5 g, range: 10.3–112.1 g) and the shortest fish in our sample (x¯ = 13.3 cm, range: 9.7–19.6 cm). Females were intermediate, falling between the two male ARTs in body mass (x¯ = 52.0 g, range: 11.4–124.1 g) and length (x¯ = 16.3 cm, range: 11.0–21.3 cm; see Appendix S2). At age 3 (the youngest age class at which the two tactics could be compared), the two male types did not differ significantly from one another in body length (Figure 4; permutation *t*‐test; *p* = 0.72), but by age 4 and older, guarder males were consistently larger than sneaker males (Figure 4; permutation *t*‐tests; *p* < 0.02 for all). Guarder males were also significantly larger than females beginning at age 4 (permutation *t*‐tests; *p* < 0.01 for all ages).

**FIGURE 5:**
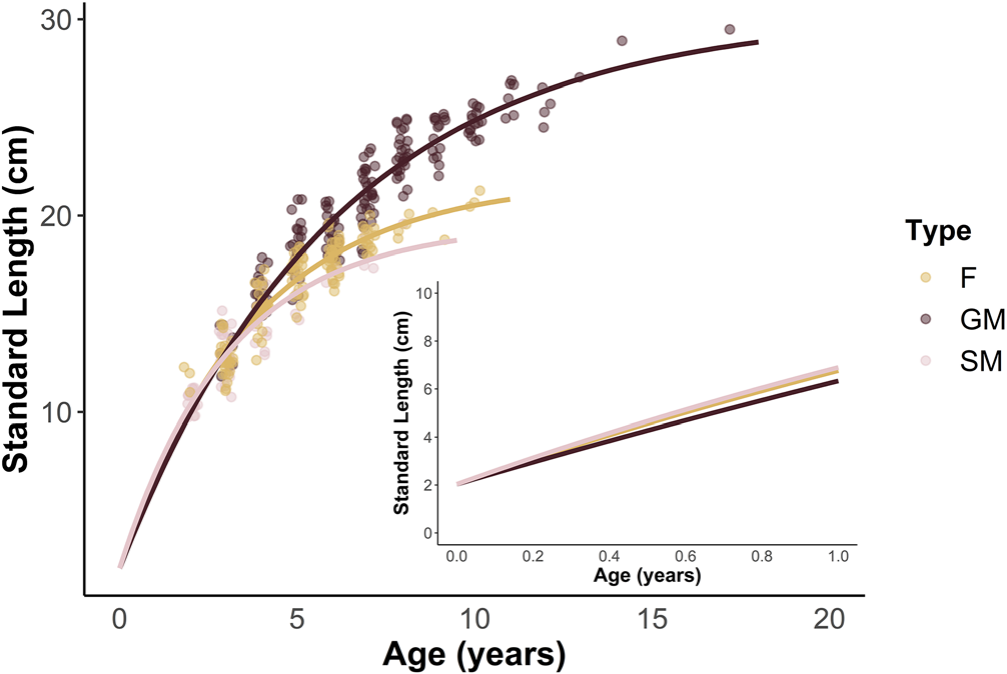
von Bertalanffy growth functions plotted for females (F, *n* = 138), guarder males (GM, *n* = 176) and sneaker males (SM, *n* = 57). The function for females is *L*_*t*_ = (21.8 − (21.8–2.035)*e* ^−0.273*t^. The function for guarder males is *L*_*t*_ = 30.3 − (30.3–2.035)*e* ^−0.165*t^. The function for sneaker males is *L*_*t*_ = (19.5 − (19.5–2.035)*e* ^−0.325*t^. Functions are only shown extrapolated until the A_0.95_ of each particular group. Inset shows the detailed extrapolated growth curves over the first year of life. Data points are jittered slightly to improve visibility.

Our estimated growth functions (VBGF, see Methods and Table 1) implied that females and sneaker males in the first year of life were larger than guarder males (Figure 5). The crossover point on the VBGF curves, after which sneaker males are smaller than guarder males, occurred at 2.8 years. This point corresponds with our permutation *t*‐test results, where the sizes of sneaker males and guarder males were not statistically different at age 3 (Figure 4). Females and sneaker males also differed from one another in terms of growth rate parameters, with females having a slower growth rate coefficient [Δx̄ ± standard error (SE) = −0.054 ± 0.001; permutation *t*‐test: *p* < 0.001], while also having a larger *L*_∞_ value (Δx̄ ± SE = 2.305 ± 0.057; permutation *t*‐test: *p* < 0.001) compared to sneakers.

**TABLE 1:** von Bertalanffy growth function parameters [± standard error (SE)] for *Porichthys notatus* sampled from intertidal breeding grounds.

Parameter	Female	Guarder male	Sneaker male
*L*_0_ (in cm)	2.035 ± 0.05	2.035 ± 0.05	2.035 ± 0.05
L_max_ (in cm)	21.3	29.5	19.6
*L*_∞_ (in cm)	21.8 ± 0.41	30.3 ± 0.58	19.5 ± 0.72
k (unitless)	0.27 ± 0.01	0.17 ± 0.01	0.33 ± 0.03
t_0_ (in years)	−0.37 ± 0.01	−0.42 ± 0.03	−0.30 ± 0.02
A_0.95_ (in years)	11.3 ± 0.02	17.7 ± 0.05	8.9 ± 0.03

*Note:* *L*_0_ is the length at t = 0, which is the time of young‐of‐the‐year (YOY) detachment in our model, L_max_ is the largest individual of that type encountered, *L*_∞_ is the asymptotic maximum length, k is the growth rate parameter, t_0_ is the age a fish would be a length of 0 and A_0.95_ is longevity as calculated in Taylor (1958).

The VBGFs themselves fit the data well with R^2^_adj_ values of 84.5% for sneaker males, 86.8% for females and 90.0% for guarder males, indicating that the growth of these midshipman were accurately described by the growth functions. Having specific VBGFs differentiated by midshipman type also improved overall model fit (likelihood ratio test; χ^2^ = 257.8, *p* < 0.001), indicating that these age‐growth relationships were best analysed by sex or male tactic. Based on our bootstrap results, both the growth rate parameter k and the maximum length *L*_∞_ were significantly different across all three groups (PERMANOVA: *p* < 0.001 for all comparisons for both; Appendices S3 and S4). In pair‐wise comparisons, females had significantly higher growth coefficient (Δx̄ ± SE = 0.108 ± 0.002; permutation *t*‐test: *p* < 0.001), and a significantly lower *L*_∞_ value than guarder males (Δx̄ ± SE = 8.464 ± 0.190; permutation *t*‐test: *p* < 0.001). Likewise, sneaker males had significantly higher growth rates (Δx̄ ± SE = 0.161 ± 0.004; permutation *t*‐test: *p* < 0.001) and significantly lower *L*_∞_ values (Δx̄ ± SE = 10.769 ± 0.242; permutation *t*‐test: *p* < 0.001) compared to guarder males. Weight‐length relationships also differed between guarder males, sneaker males and females (likelihood ratio; χ^2^ = 30.8, *p* < 0.001). Females had more of an isometric growth relationship when compared to either guarder males or sneaker males, both of which had strong, positive allometric growth curves (Appendix S4). Based on the VBGFs calculated, the predicted size of guarder males, sneaker males and females at age 1 should have been 6.33 cm [95% confidence interval (CI): 6.16–6.50 cm], 6.90 cm (95% CI: 6.65–7.15 cm) and 6.76 cm (95% CI: 6.61–6.91 cm), respectively. Although the predicted mean size at age 1 of guarder males and females fits within the 95% CI of known age 1 juveniles from the wild (95% CI: 6.11–6.81 cm), the predicted mean size of sneaker males does not, though the 95% CIs do overlap.

**FIGURE 6:**
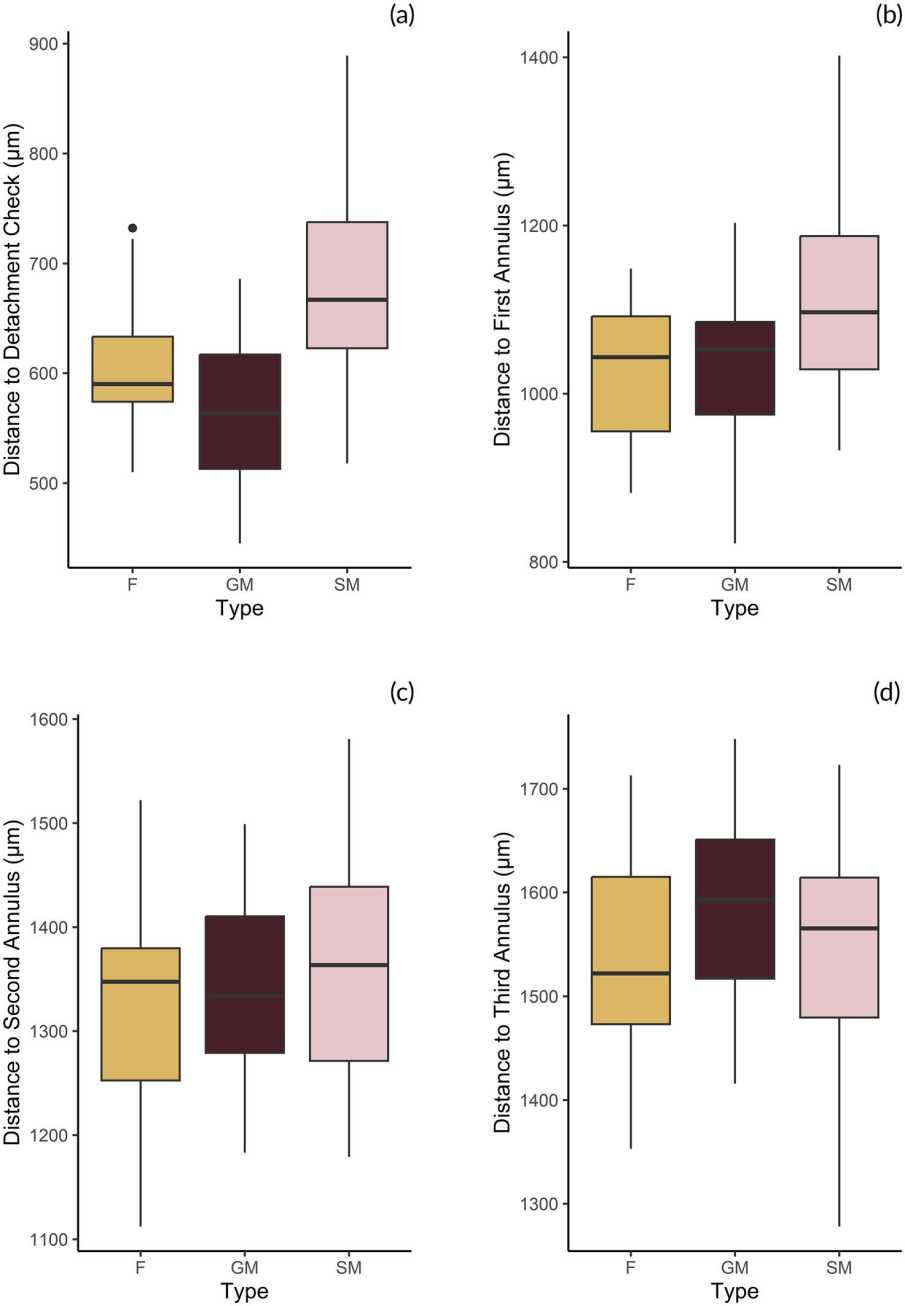
Distance measured along the ventral midline of the otoliths of females, sneaker males and guarder males to the start of the detachment check (a) and first three annuli (b–d). A total of 22 females, 30 sneaker males and 23 guarder males were included in these comparisons.

Midshipman differed from one another in terms of distance to the detachment check mark (PERMANOVA: F_2,74_ = 14.491, *p* < 0.001; Figure 6a). Sneaker males had a greater distance to the detachment check mark on their otolith compared to guarder males (permutation *t*‐test: Δx̄ ± SE = 92.452 ± 33.411; *p* < 0.001). Sneakers also had a greater distance to the first detachment check than females (permutation *t*‐test: Δx̄ ± SE = 64.408 ± 25.866; *p* = 0.008). Guarders did not differ from females in terms of distance to the detachment check (permutation *t*‐test: Δx̄ ± SE = 27.985 ± 22.710; *p* = 0.208). The distance to the first annulus also differed between the three midshipman types (PERMANOVA: F_2,74_ = 6.097, *p* = 0.003), with sneaker males having the greatest distance to the first annulus on average (Figure 6b). Sneaker males had a greater distance to the first annulus on their otolith compared to guarder males (permutation *t*‐test: Δx̄ ± SE = 108.120 ± 33.058; *p* < 0.001). Sneakers also had a greater distance to the first annulus than females (permutation *t*‐test: Δx̄ ± SE = 71.736 ± 27.296; *p* = 0.007). Guarders did not differ from females in terms of distance to the first annulus (permutation *t*‐test: Δx̄ ± SE = 41.636 ± 28.730; *p* = 0.111). However, the distance to the second did not differ between sneakers, guarders and females (PERMANOVA: F_2,72_ = 0.700, *p* = 0.513; Figure 6c) nor the third annulus (PERMANOVA: F_2,65_ = 1.414, *p* = 0.228, Figure 6d).

## 4 DISCUSSION

We aged plainfin midshipman adults using the break‐and‐burn method on their sagittal otoliths and used these ages to characterize the age‐growth functions of each sex and male ART. Guarder males were on average the oldest plainfin midshipman sampled (mean of 7.0 years old and an estimated longevity of 17.7 years). In contrast, sneaker males were, on average, the youngest fish at our study sites (mean of 3 years and an estimated longevity of 8.9 years). Females were intermediate in both size and age (mean of 5.1 years and an estimated longevity of 11.3 years). All three categories of midshipman fish showed different VBGF growth trajectories, with bootstrap analyses indicating that the growth parameter, k, and the asymptotic length, *L*_∞_, differed significantly across plainfin midshipman male tactics and sexes.

The presence of consistent and clear annuli at regular distances made them straightforward to discern from surrounding checks and check marks and useful for ageing. The first annulus sometimes appeared quite close to the detachment check, and the increment size likely related to how much the plainfin midshipman grew in the first year of life, and probably this was related to how late in the season they detached from the rock. We also used the known‐age juvenile individuals to test how well our VBGFs predicted size at age 1. The fact that our estimated growth functions fit the actual body size data so well for females and both male tactics (Figure 5), and that the outputs of the VBGFs mostly matched the observed sizes of age 1+ midshipman, provides strong support for the accuracy of our ageing technique.

The plainfin midshipman fish has a fairly unique natural history and reproductive ecology. After leaving their nests, plainfin midshipman juveniles use eelgrass beds near their natal intertidal zones as nursery habitats (Heck Jr. et al., 2003; Proudfoot et al., 2023), potentially remaining there for the first year of life (Andy Lamb, personal communication, Micah Quindazzi, 11 August 2022, personal observations). Eventually these fish move to deeper parts of the ocean where they can be regularly caught by deep‐sea otter trawls (Ibara et al., 1983; Sisneros, 2007). After sexual maturation, they undergo potentially annual spring migration from the deep back to the rocky intertidal zone. After a protracted reproductive season from late April to late July/August, they return to depth (Arora, 1948; Hubbs & Schultz, 1939). Guarder males care for offspring for a 2‐ to 3‐month period in excavate nesting burrows beneath intertidal rocks; during this paternal care period, they fast, undergo regular air emersion and lose body condition (Bose et al., 2014, 2016; Cogliati et al., 2015). The extensive vertical migrations, regular low‐tide emersion and long‐duration parental care could have generated otolith checks and check marks observed (Buckmeier & Smith, 2020; Campana, 1989); however, more research is needed to verify this idea. As adults, guarder males continue to grow larger, whereas sneaker males appear to plateau in comparison. Comparisons of diets outside of the spawning season and assessments of the start and periodicity of reproductive events within these fish could illuminate the causes behind these differences. It is possible that based on sheer size, guarder males are able to access larger food sources than sneakers, or are at colder temperatures (greater depth), and thus more growth can be directed towards maintaining a larger overall size.

Although our ageing technique was enabled by the observations of known‐age juvenile data, we were limited by the fact that we could not identify the sex of these individuals aged 1 year or younger. The predicted size of guarder males and females were within the 95% CI of the size at age 1+ of midshipman, whereas the predicted mean size of sneaker males did not, though the overall 95% CIs were overlapping. Moving forward, our ageing technique could be further bolstered through an extensive validation effort via tagging or longer‐term rearing, which are common methods of age validation (Hüssy et al., 2019).

Otolith ageing has been previously used to identify sexual dimorphism in life histories within the Batrachoididae family through VBGFs (Table 2) (Malca et al., 2009; Palazón‐Fernandez et al., 2010; Radtke et al., 1985). However, our study is the first to extend this concept to male ARTs within this family. A previous study by Bass et al. (1996) used daily ring counts on otoliths to age young (1 year old) plainfin midshipman, but growth has not been studied before in this species. The 1‐year‐old fish in the Bass et al. (1996) study were estimated to be much younger than similar‐sized fish in our study. One possibility for this difference is that we counted annuli, whereas Bass et al. (1996) counted daily rings. Daily ring deposition in juvenile plainfin midshipman has been verified in laboratory settings (Campana, 1984), but beyond ~100 days of age, daily rings become challenging to read, potentially obscuring estimates (Brothers et al., 1976; Campana & Neilson, 1985; Campana & Thorrold, 2001; Dicenzo & Bettoli, 1995; Gauldie & Nelson, 1990; Katayama, 2018; Sakaris & Irwin, 2008). Daily ring methods are especially useful for species that live in stable resource environments, such as fast‐growing tropical fish (Brothers et al., 1976). Some authors have suggested that the break‐and‐burn annular ring reading is a more effective way to age species that encounter seasonal growth discontinuities, such as slow‐growing temperate fish (Beckman & Wilson, 1995) like plainfin midshipman. In is worth noting that the southern population of midshipman studied in Bass et al. (1996) are known to be smaller (Cogliati et al. 2014), and thus these fish could have indeed been younger.

**TABLE 2:** A list of von Bertalanffy growth function parameters (*L*_∞_ and k) and longevity [A_0.95_, based on Taylor (1958)] of Batrachoid fishes from various studies, including the current study.

Species	Type	*L*_∞_ (cm)	k	A_0.95_ (years)	*n*	Reference
*Opsanus tau*	Male	40.8	0.15	19.6	41	Radtke et al. (1985)
*O. tau*	Female	27.2	0.39	7.2	20	Radtke et al. (1985)
*Opsanus beta*	Male	39.4	0.30	10.3	24	Malca et al. (2009)
*O. beta*	Female	20.1	0.79	4.3	20	Malca et al. (2009)
*Halobatrachus didactylus*	Male	47.7	0.15	19.4	326	Palazón‐Fernandez et al. (2010)
*H. didactylus*	Female	36.4	0.20	14.2	345	Palazón‐Fernandez et al. (2010)
*Colletteichthys dussumieri*	Male	35.1	0.17	17.0	199	Sebastian (2011)
*C. dussumieri*	Female	30.3	0.22	13.2	152	Sebastian (2011)
*Porichthys notatus*	Guarder male	30.3	0.17	17.7	176	This study
*P. notatus*	Sneaker male	19.5	0.33	8.9	57	This study
*P. notatus*	Female	21.8	0.27	11.3	138	This study

There was overlap in both size and age for sneakers and guarders, with 74% of males (70% of sneakers and 76% of guarder males) being between the ages 3 and 8. The oldest sneaker males in our study sample were larger than 3‐ and 4‐year‐old guarder males (Figure 4). So, despite having a body size that would be sufficient to hold a nest (as a guarder male would), these large sneakers still had internal and external traits (golden belly, extremely large testes, small accessory glands, underdeveloped sonic muscles and conical swim bladders) (Bass & Marchaterre, 1989; Bass et al., 1996; Fitzpatrick et al., 2016; Miller et al., 2019; Mohr et al., 2017; Rogers et al., 2023). Our data indicate that sneaker males migrate to the breeding grounds earlier in their lives than guarder males and maintain sneaker behaviour, morphology and anatomy even after surpassing the minimum guarder male body size. This supports prior findings (Bass et al., 1996). Similarly, in other fish species, sneaker males appear on the breeding grounds earlier than guarder males, indicating they have early sexual maturation (Engqvist & Taborsky, 2016; Taborsky, 2008).

In addition to substantial overlap in ages and body sizes and different growth trajectories, there are several additional reasons why we believe that sequential transition from one tactic to the other is highly unlikely. First, transitioning from a sexually mature sneaker male to a guarder male (or vice versa) would require major neuroanatomical, physiological, vocal‐motor system, gonadal and swim bladder remodelling, which would be highly costly, especially after recent investment in tactic‐specific structures. These ARTs differentiate during the transition to sexual maturity (Bass et al., 1996; Grober et al., 1994). Second, after 20+ years of conducting field observations on the plainfin midshipman fish, we have never observed a male that is seemingly in transition between two tactics. Guarder males that are presumed to be unsuccessful on the spawning grounds do conduct opportunistic parasitic spawning, but they lack any of the sneaker‐specific adaptations (Lee & Bass, 2004, 2006).

Our estimated growth functions predicted that sneaker plainfin midshipman males would be larger than guarder males at ages 1 and 2. The physical measurements of annuli distances corroborated this greater initial growth for sneaker males compared to guarder males at both age 1 and detachment, with guarder males catching up and surpassing sneakers’ body size by age 3. Sneaker males appear to grow faster and sexually mature earlier, whereas guarder males appear to delay sexual maturation (at least until age 3) and invest their energy into survival and growth. Bass et al. (1996) suggested that the ‘decision’ by males to adopt either a guarder or sneaker growth trajectory is determined early in life, and our data are consistent with their conclusion. It remains to be seen whether this fixed‐tactic expression is genetically inherited or conditionally determined, though one study suggests that tactic expression can be, in part, influenced by rearing density, which would imply that these tactics are conditionally determined (Foran, 1998).

Our results strongly imply that plainfin midshipman fish guarder and sneaker males represent two separate fixed developmental trajectories. Based on a large sample spanning the full‐size range of midshipman adults captured from intertidal breeding grounds, we have shown that guarder and sneaker males likely represent fixed tactics with distinct developmental trajectories. Sneaker males grow faster initially, but guarder males grow to an overall larger size and live for twice as long. Sexual dimorphism in the age‐growth relationship is present in plainfin midshipman, which is in line with other species in the Bathrachoidae family. Follow‐up research should consider the degree to which these tactics are parentally inherited or are environmentally induced by early‐life conditions. Assessing the fitness of each ART by size and age would improve our understanding of the trade‐offs between these two ARTs. Our study provides researchers with a clear way to age plainfin midshipman and provides an age‐growth relationship for adults, which is an important prerequisite in the studies of the life histories and population ecology of this and other species.

## AUTHOR CONTRIBUTIONS

Micah J. Quindazzi: ideas, data analysis, lead author on manuscript preparation and funding. Angus Townsend: data generation, data analysis, manuscript preparation and funding. Aneesh P.H. Bose: ideas, data analysis, sample collection, manuscript preparation and funding. Sigal Balshine: ideas, sample collection, manuscript preparation and funding. Francis Juanes: manuscript preparation and funding.

## FUNDING INFORMATION

Micah J. Quindazzi was funded through a Mitacs fellowship and NSERC CGS‐D. Angus Townsend received an NSERC USRA award; Francis Juanes was funded through NSERC DG and the Liber Ero Foundation. Sigal Balshine was funded through an NSERC DG grant. Aneesh P.H. Bose was supported by the Swedish Research Council (Vetenskapsrådet, DNR 2023‐03866). The authors declare no competing interests that would bias this work.

## Supporting information


**Data S1.** Supporting information.
