# Supplementary material for: Growth and age differences between two male alternative reproductive tactics in the plainfin midshipman
Source: J Fish Biol. 2025 Nov 17;108(2):707–17. doi: 10.1111/jfb.70274 (PMC13052468; doi:10.1111/jfb.70274)
Supplement: Supplementary file 1 — Data S1. Supporting information. [file JFB-108-707-s001.docx]

**Appendix**

**Appendix 1**. Location information (latitude and longitude) for the 374 samples collected for this study, including sex and ART.

| **Location Name** | **Latitude** | **Longitude** | **Guarder Males** | **Sneaker Males** | **Females** |
| --- | --- | --- | --- | --- | --- |
| Crescent Beach | 49°04’N | 122°88’W | 48 | 19 | 42 |
| Ladysmith Inlet | 49°01’N | 123°46’W | 25 | 0 | 6 |
| Lantzville | 49°15’N | 124°04’W | 10 | 0 | 0 |
| Seal Rock | 47°43’N | 122°53’W | 1 | 2 | 2 |
| Transfer Beach | 48°59’N | 123°48’W | 94 | 36 | 89 |


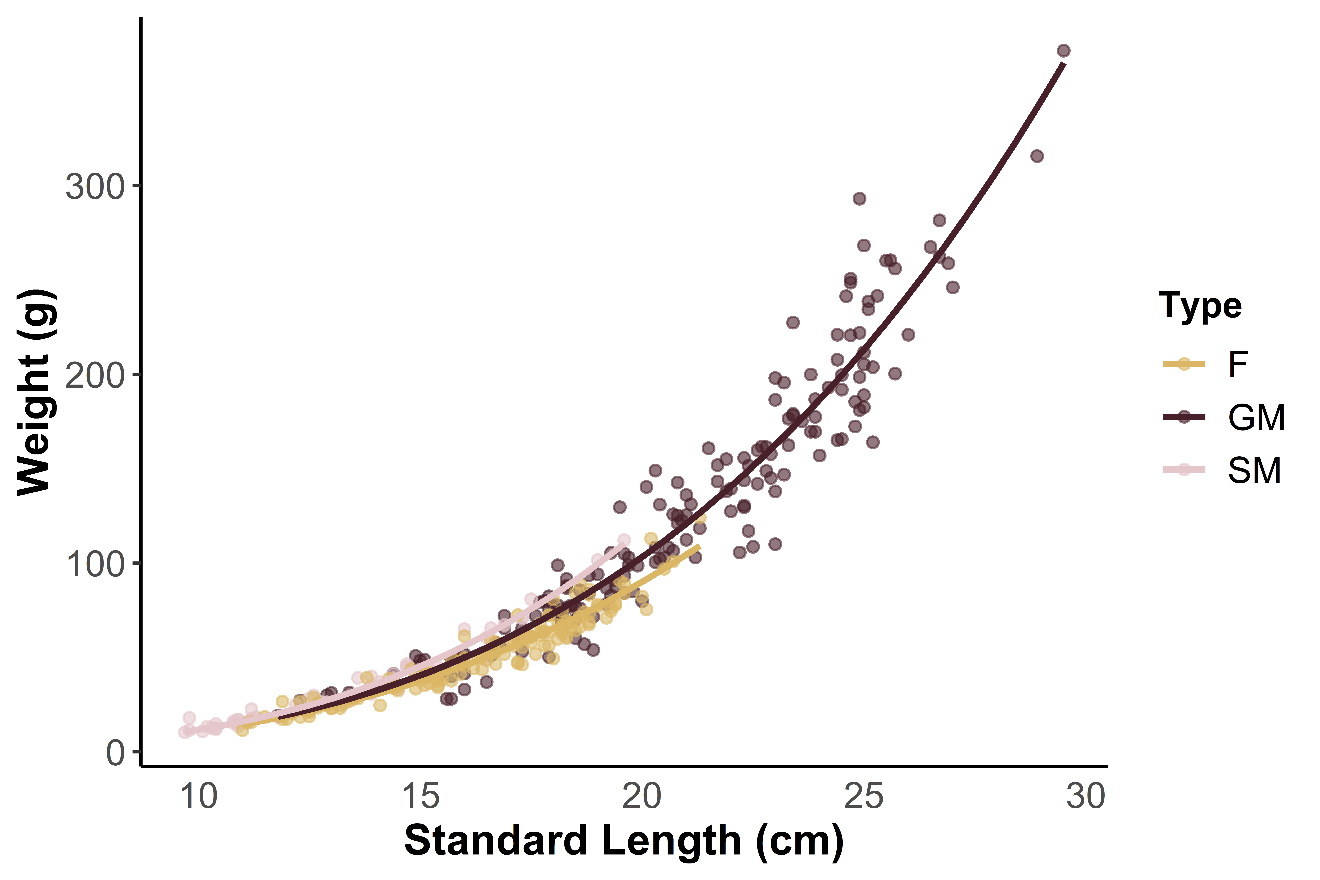


**Appendix 2**. Scatterplot showing the relationship between the standard length and weight of *P. notatus* (n = 371) separated into guarder males (GM, n = 176), sneaker males (SM, n = 57), and females (F, n = 138). The relationship between weight and length for guarder males was y = 0.0061x^3.25^ (R^2^ = 92.2%). The relationship between weight and length for sneaker males was y = 0.0055x^3.33^ (R^2^ = 97.7%). The relationship between weight and length for females was y = 0.0100x^3.04^ (R^2^ = 92.9%).


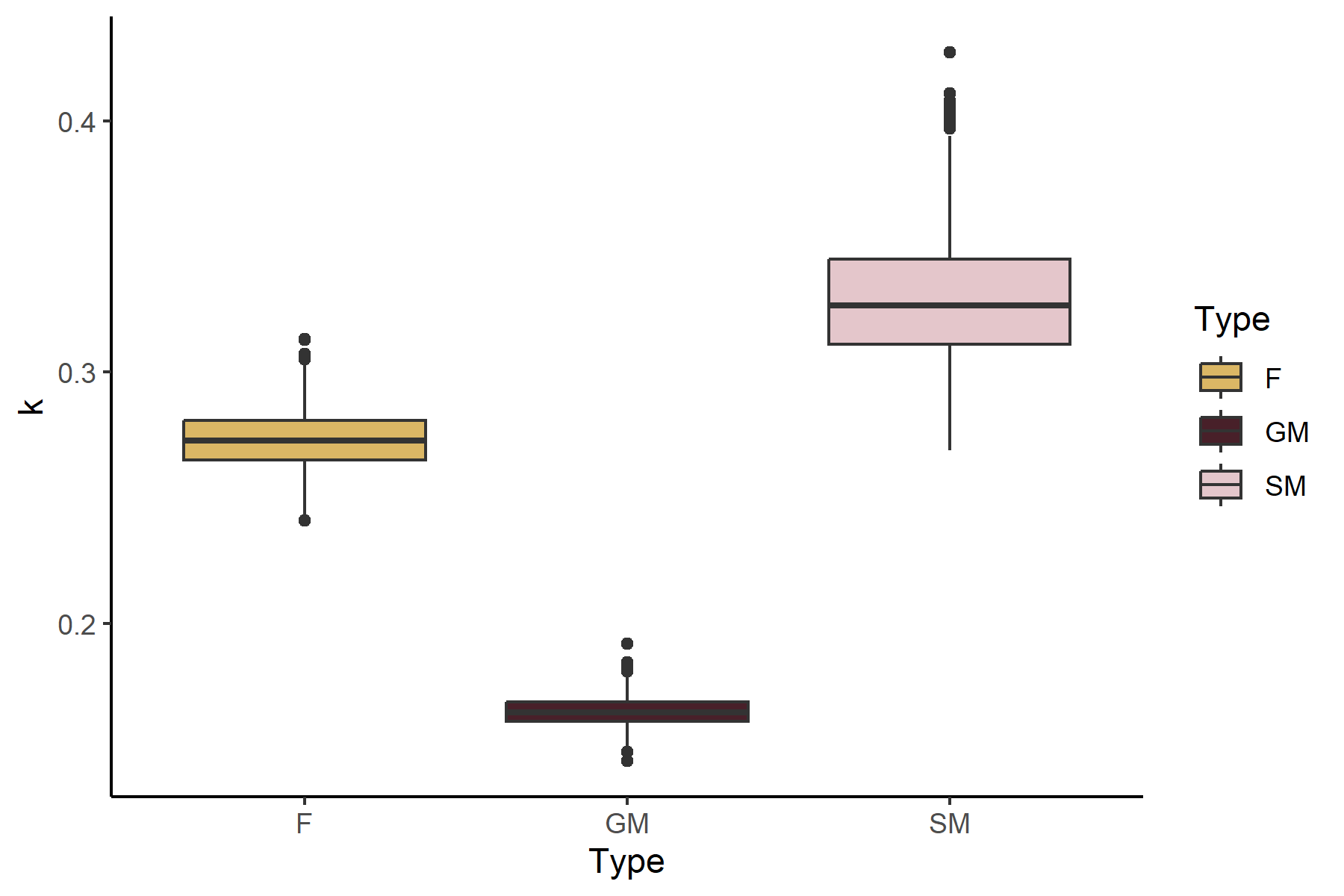


**Appendix 3.** Boxplots showing the bootstrapped values for the growth coefficient, k, for females, guarder males, and sneaker males. 1,000 bootstraps were conducted. See methods for details.


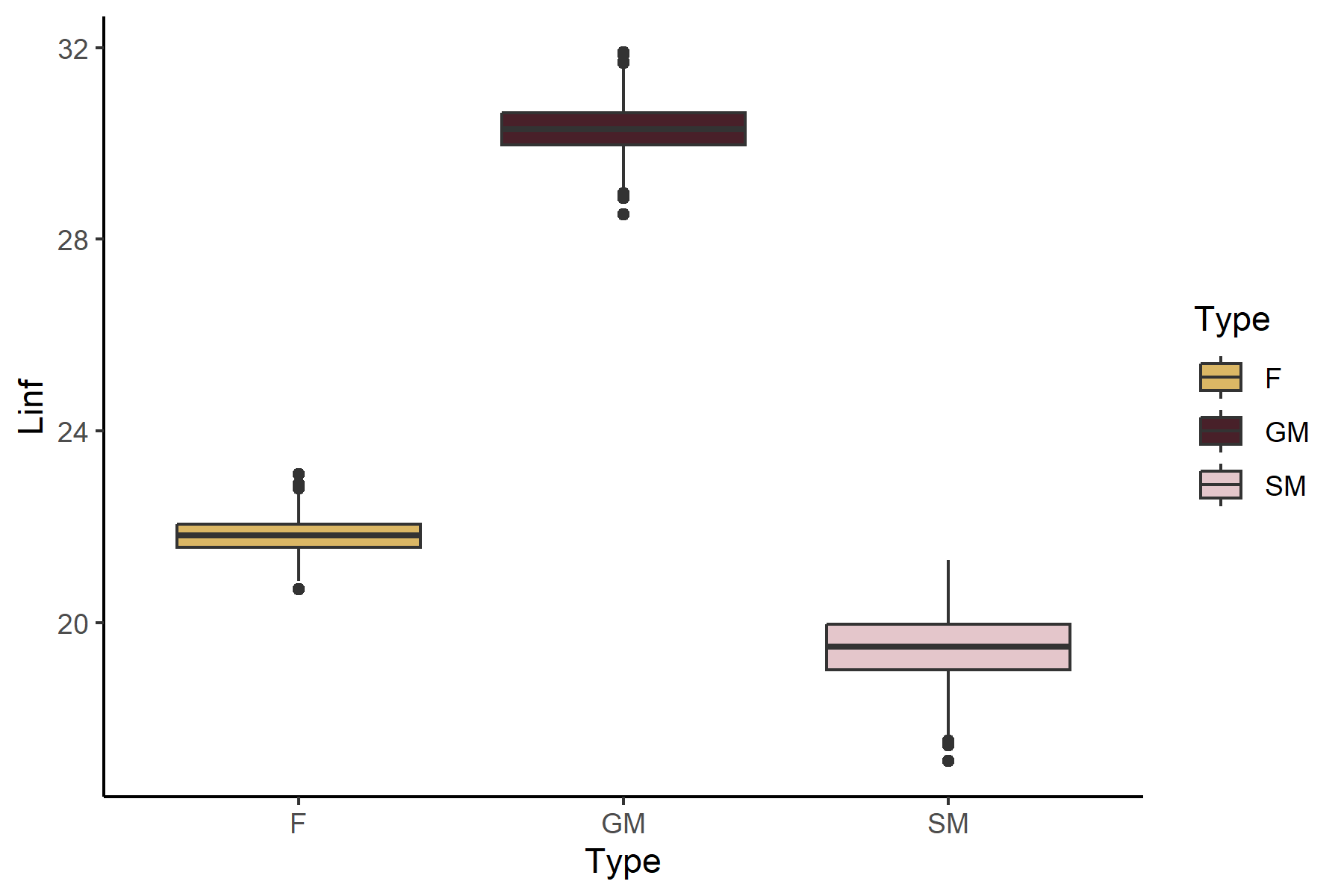


**Appendix 4.** Boxplots showing the bootstrapped values for the asymptotic maximum length, Linf, for females, guarder males, and sneaker males. 1,000 bootstraps were conducted. See methods for details.
